# Cognition, affect, and learning aspirations in public health development history education: cross-sectional associations in a large sample of university students in Eastern China

**DOI:** 10.3389/fpubh.2026.1921511

**Published:** 2026-08-27

**Authors:** Shiyu Zhang

**Affiliations:** School of Public Administration, Hangzhou Normal University, Hangzhou, Zhejiang, China

**Keywords:** cognition–affect–conation framework, educational cognition, educational emotion, ideological and political education, public health development history, public health learning aspirations

## Abstract

**Introduction:**

Historically grounded narratives are increasingly incorporated into values-oriented higher education, yet limited empirical research has examined how university students’ appraisals of public health development history are associated with their affective responses and stated learning demands. Guided by the Cognition–Affect–Conation (CAC) framework as a conceptual organizing framework, this study examined the cross-sectional associations among Educational Cognition, Educational Emotion, and Public Health Learning Aspirations (PHLA) within Ideological and Political Education (IPE) in Chinese universities. PHLA was used as an umbrella term for two related forms of stated educational demand: Demand for Educational Resources (DER) and Demand for Educational Content (DEC).

**Methods:**

A cross-sectional online survey was administered in September 2025 to 1,142 university students recruited through stratified cluster sampling; 92.2% attended institutions in Eastern China. From a self-developed 33-item questionnaire, 24 substantive Likert-type items measuring Educational Cognition, Educational Emotion, DER, and DEC on five-point scales were analyzed. Descriptive statistics, Spearman rank-order correlations, and bootstrap cross-sectional indirect-association models were estimated using PROCESS Macro Model 4 for SPSS with 5,000 resamples.

**Results:**

All four study variables had means above 4.0, with Educational Emotion showing the highest mean (*M* = 4.528, SD = 0.589). The variables were positively intercorrelated (*ρ* = 0.649–0.826, all *p* < 0.01), supporting H1. In the specified cross-sectional regression models, Educational Cognition was positively associated with Educational Emotion (*β* = 0.65), DER (*β* = 0.56), and DEC (*β* = 0.45; all *p* < 0.001), supporting H2. The indirect association involving Educational Emotion was statistically different from zero for DER (estimate = 0.20, 95% bootstrap CI [0.15, 0.23]; 26% of the total association) and DEC (estimate = 0.28, 95% bootstrap CI [0.23, 0.33]; 39% of the total association). These estimates came from separate outcome models and were not directly compared.

**Discussion:**

The findings describe the co-occurrence of educational appraisal, affective-motivational response, and stated learning demand, a pattern compatible with the CAC framework but not evidence of temporal mediation or causality. By distinguishing substantive content demand from resource-related demand, the study provides a preliminary basis for examining how public health development history is appraised within value-laden education. The findings may inform the development of historical content and supporting resources in IPE and medical humanities curricula. Interpretation remains limited by self-report measurement, possible socially desirable responding, operational overlap among the study-defined constructs, and the concentration of the sample in Eastern China.

## 1 Introduction

### 1.1 Public health as a context for value-laden education in China

Over the past several years, recurrent infectious outbreaks, the advancement of the Healthy China 2030 agenda, and growing concerns over health equity have collectively repositioned public health beyond its clinical and technical boundaries. It has become a sphere in which national identity, collective solidarity, individual values, and population behavior are closely interrelated (1). As public health increasingly operates as a domain of shared values rather than purely technical practice (2, 3), attention to value-laden education is particularly relevant for university students preparing for public health responsibilities (4).

Within the Chinese higher education system, Ideological and Political Education (IPE) is recognized as an institutionalized form of value-laden education with strong local features (5). Prior empirical work has reported associations of IPE-related measures with psychological well-being (6), national identification (7), and competence development (8). These studies support closer examination of educational resources and psychological correlates within IPE.

### 1.2 Research gap: public health development history as an underexamined IPE resource

However, empirical research on IPE in Chinese higher education has concentrated on a relatively narrow range of educational resources, including curriculum-based ideological instruction (9, 10) and digitally mediated educational approaches (11, 12). Parallel research in health-professions education has examined the historical development of public health education, as well as the inclusion of historical pandemics, medical humanities, and historical approaches in teaching (13–16). Nevertheless, public health development history has rarely been investigated as a structured, value-laden resource within IPE.

Public health development history refers to the historical record of how societies have organized collective efforts to prevent disease and promote health, reflecting historical shifts in emphasis across community living, environmental sanitation, and disease control (17). Recent studies have described historical perspectives as relevant to understanding public health values, institutional development, health inequities, structural biases, and contemporary public health practice (18, 19). Related research has also addressed public health governance values, expectations for public health education after COVID-19, and the educational use of past pandemics (20–22). Yet little is known about how students’ appraisals of public health development history are associated with their emotional engagement and Public Health Learning Aspirations (PHLA) within IPE.

A recent study of public trust in AI-enabled telemedicine conceptualized cognitive and affective responses as interrelated dimensions of health-related perceptions (23). Despite the substantive difference between health technology and public health history, this work offers a contemporary conceptual parallel for examining Educational Cognition and Educational Emotion jointly, without imposing a temporal or causal ordering between them.

### 1.3 Theoretical framework and research hypotheses

To frame these relationships, the current study draws on the Cognition–Affect–Conation (CAC) framework (24, 25), which distinguishes cognitive, affective, and conative components of human psychological functioning. Educational Cognition refers to students’ rational appraisal of the national narratives, institutional realities, and values embedded in public health development history; Educational Emotion denotes affective engagement associated with this material; and Public Health Learning Aspirations (PHLA) denote students’ future-oriented willingness to engage further with public-health-related learning. PHLA was used as an umbrella term for two related but separately analyzed forms of stated educational demand: Demand for Educational Resources (DER) and Demand for Educational Content (DEC), with detailed conceptual definitions provided in the Measures section. The CAC framework guides the organization of the variables but does not establish their temporal or causal order in this cross-sectional study.

Building on this theoretical foundation, the present study aimed to examine (a) the association between Educational Cognition regarding public health development history and PHLA among the surveyed student cohorts and (b) whether Educational Emotion was statistically implicated in the cross-sectional indirect association between these variables. The analysis was intended to extend the descriptive application of the CAC framework to value-laden historical education within IPE in the sampled institutions, predominantly in Eastern China.

Guided by the above framework, this study sets out a group of hypotheses about the relationships among Educational Cognition, Educational Emotion, and PHLA in university students. Accordingly, the following hypotheses are proposed:

*H1*: Educational Cognition, Educational Emotion, DER, and DEC are all positively correlated with one another.

*H2*: In the specified cross-sectional regression models, Educational Cognition is positively associated with Educational Emotion, DER, and DEC.

*H3*: Educational Emotion is statistically implicated in the cross-sectional indirect association between Educational Cognition and PHLA.

*H3a*: Educational Emotion is statistically implicated in the cross-sectional indirect association between Educational Cognition and DER.

*H3b*: Educational Emotion is statistically implicated in the cross-sectional indirect association between Educational Cognition and DEC.

## 2 Materials and methods

### 2.1 Data source and participants

Data were collected in September 2025 through a cross-sectional online questionnaire survey. A stratified cluster sampling strategy was used to obtain variation in academic disciplines, institutional types, and student backgrounds within the surveyed cohorts. The questionnaire recorded institution type and broad geographic region, but not university names; therefore, the specific participating universities cannot be identified from the dataset.

A total of 1,274 questionnaires were distributed, and 1,142 valid questionnaires were retained after data screening, yielding an effective response rate of 89.6%. Of the analytic sample, 9.2% attended Double First-Class universities, 86.7% public undergraduate institutions, 3.2% private undergraduate institutions, and 0.9% higher vocational colleges. Most respondents (92.2%) attended institutions in Eastern China; the remainder were distributed across South, Central, North, Northwest, Southwest, and Northeast China. A complete list of the 33 questionnaire items, together with their response formats, is provided in Supplementary Table S1.

This study involving human participants received retrospective ethics-compliance review from the Ethics Committee of the School of Public Administration, Hangzhou Normal University (Review No. 20260009). The Committee reviewed the study documentation, including the participant informed-consent materials, in accordance with applicable ethical principles and the Declaration of Helsinki. All participants provided electronic informed consent before completing the anonymous questionnaire. As the ethics review was conducted after data collection, it should not be interpreted as prospective ethical approval.

### 2.2 Measurement of variables

The full survey instrument contained 33 items, comprising nine demographic and background questions and 24 substantive Likert-type items. These substantive items comprised four items measuring Educational Cognition, five measuring Educational Emotion, five measuring DEC, and 10 measuring DER. All items used five-point response scales scored from 1 to 5.

#### 2.2.1 Educational cognition

Educational Cognition assessed students’ evaluations of the performance of Ideological and Political Education using the history of public health development. The measure comprised four items evaluating: (1) content authenticity, (2) emotional appeal, (3) capacity for value guidance, and (4) practical inspiration. Each item was rated on a five-point scale ranging from 1 (“very poor”) to 5 (“very good”), with higher scores indicating a more favorable comparative evaluation.

#### 2.2.2 Educational emotion

Educational Emotion was measured using five items assessing students’ affective responses, value endorsement, and behavioral intentions concerning the history of China’s public health development. The items addressed: (1) whether studying this history helps strengthen national pride and national identification; (2) whether the patriotic spirit embedded in this history is worthy of being inherited and promoted; (3) whether universities should incorporate this type of historical education as an important component of IPE; (4) willingness to participate in public-health-related volunteer services, such as health education and free-clinic activities; and (5) pride in the achievements of China’s public health enterprise. Responses were recorded on a five-point scale ranging from 1 (“strongly disagree”) to 5 (“strongly agree”). Higher mean scores indicated stronger endorsement of these affective and value-related statements.

#### 2.2.3 Public health learning aspirations

The PHLA contains two study-defined dimensions: DEC and DER, which were calculated and analyzed separately. All items were rated on a five-point agreement scale ranging from 1 (“strongly disagree”) to 5 (“strongly agree”).

The DEC was measured using five items concerning the content of school courses or educational activities. The items assessed whether: (1) courses or activities emphasized identifying the IPE elements embedded in the history of public health development; (2) the development of public health in China was systematically introduced across different historical periods; (3) institutional, scientific and technological, and organizational innovations achieved during public health crises were presented; (4) the contributions of diverse actors, including scientists, frontline healthcare workers, public health administrators, and community workers, were covered; and (5) educational content remained coherent and consistent across different courses or activities. Higher mean scores indicated stronger endorsement of the corresponding educational-content statements.

The DER was measured using 10 items covering educational resources, instructional support, and delivery conditions. The items assessed whether: (1) the institution provided sufficient media resources, such as documentaries, courseware, and historical footage; (2) the institution collaborated with practice bases or memorial venues, such as medical museums and “red” education bases; (3) front-line public health practitioners, including CDC personnel and healthcare workers involved in epidemic response, were invited to share their experiences; (4) new media, such as WeChat official accounts and short-video platforms, were used to publicize exemplary individuals and their deeds; (5) instructors possessed relevant disciplinary knowledge and humanistic literacy and could explain historical events and their underlying values in depth; (6) educational formats were diverse, including thematic lectures, film-viewing seminars, situational dramas, and social practice; (7) the teaching process was lively and engaging and stimulated learning interest and reflection; (8) students had opportunities to select suitable learning approaches, such as online learning, field visits, and group projects; (9) interaction and discussion were encouraged to enhance students’ sense of participation; and (10) accessible feedback channels allowed students’ views concerning institutional educational activities to be heard. Higher mean scores indicated stronger endorsement of the corresponding educational-resource and instructional-support statements.

### 2.3 Data analysis

#### 2.3.1 Demographic characteristics analysis

This study first conducted descriptive statistical analyses to examine the demographic characteristics of the respondents and clarify the sample composition. The reported variables included gender, academic year, disciplinary background, political affiliation, institution type, geographic region, receipt of financial aid, and prior visits to medicine-, anti-epidemic-, or public-health-themed exhibitions, providing a structural overview of the surveyed cohorts and a contextual basis for subsequent analyses.

#### 2.3.2 Reliability and validity analysis of the questionnaire

To ensure the scientific rigor of the measurement instrument, the reliability and validity of the questionnaire were examined prior to formal statistical analysis.

Reliability was assessed using Cronbach’s alpha coefficient to evaluate internal consistency. A commonly accepted threshold of 0.70 was used to determine acceptable reliability for both the overall scale and subscales. Values at the upper end of the observed range were interpreted cautiously because very high alpha coefficients may also reflect item redundancy or overlapping content.

Structural validity was examined using exploratory factor analysis (EFA) to identify the underlying dimensions and assess item loadings; loadings of 0.50 or above were treated as acceptable.

Potential common method variance was assessed using Harman’s single-factor test. All 24 substantive measurement items were entered simultaneously into an unrotated exploratory factor analysis, and the percentage of variance accounted for by the first factor was compared with the 50% diagnostic threshold. The first factor accounted for 64.61% of the total variance. To assess the discriminant validity among Educational Cognition, Educational Emotion, DEC, and DER, and to further address potential concerns about common method bias, confirmatory factor analysis (CFA) was conducted using SPSSAU. Maximum likelihood estimation was used, and discriminant validity was evaluated by comparing the model-fit indices of alternative factor structures.

Several procedural steps were used to reduce response-related method bias. Participation was anonymous, electronic informed consent was obtained before questionnaire completion, and no directly identifying information was collected. Items assessing the focal domains were organized into separate sections and used construct-specific wording. Educational Cognition used evaluative anchors ranging from ‘very poor’ to ‘very good’, whereas Educational Emotion, DER, and DEC primarily used agreement anchors ranging from ‘strongly disagree’ to ‘strongly agree’, providing limited methodological separation. Nevertheless, all focal variables were collected from the same respondents in a single survey session; no temporal or source separation, formal counterbalancing, marker variable, or social-desirability scale was implemented.

#### 2.3.3 Correlation analysis

Following the assessment of the measurement properties, Spearman’s rank-order correlation analysis was conducted to examine the pairwise relationships among the core variables, including Educational Cognition, Educational Emotion, DER, and DEC.

Correlation coefficients and their statistical significance levels were calculated to preliminarily explore the direction and strength of associations among variables.

#### 2.3.4 Cross-sectional indirect-association analysis

To examine the pattern of associations among variables at a single point in time, cross-sectional indirect-association models were estimated. Educational Cognition, Educational Emotion, and learning aspirations were entered in an ordered statistical model solely to estimate directional associations specified by the CAC framework. This analytic ordering does not demonstrate temporal precedence, causal influence, or mediation unfolding over time.

Regression-based analyses were conducted using the PROCESS macro. Bias-corrected nonparametric bootstrap estimates based on 5,000 resamples were used to evaluate the cross-sectional indirect associations. An indirect association was considered statistically different from zero when its 95% confidence interval (CI) did not include zero. These estimates quantify directional associations within the specified model at one measurement occasion and must not be interpreted as causal mediation.

#### 2.3.5 Sensitivity analyses by gender and disciplinary background

To assess whether the primary cross-sectional indirect-association patterns were robust across key demographic subgroups, the two models were re-estimated separately by gender and disciplinary background. Gender-stratified analyses were conducted separately for male and female students, with disciplinary background, institution type, and geographic region included as covariates. Discipline-stratified analyses were conducted separately for medical and non-medical students, with gender and the other prespecified demographic covariates included in the models.

Within each subgroup, Educational Cognition was specified as the focal predictor variable, Educational Emotion as the intervening statistical variable, and DER or DEC as separate outcome variables. The subgroup models used the same PROCESS Model 4 specification and 5,000 bootstrap resamples as the primary analyses. Robustness was evaluated by examining the direction and magnitude of the subgroup-specific estimates and whether the 95% bootstrap confidence intervals for the indirect effects excluded zero. These analyses were conducted as sensitivity analyses and were not treated as formal tests of between-group differences, effect modification, or moderated mediation.

## 3 Results

### 3.1 Demographic characteristics

Demographic characteristics of the 1,142 valid respondents are presented in Table 1. The majority of respondents were female (70.9%), enrolled in public undergraduate institutions (86.7%), and located in East China (92.2%). Across academic disciplines, medical-related students constituted the largest subgroup, with medicine (38.2%), engineering (19.9%), and natural sciences (13.7%) being the three most represented majors; within the medical subsample, nursing was the dominant sub-discipline (23.0% of the full sample), followed by public health and preventive medicine (8.1%). Just over half of the respondents (56.7%) reported a prior visit to a medicine-, anti-epidemic-, or public-health-related memorial or exhibition.

**Table 1:** Demographic characteristics of the respondents (*N* = 1,142).

Demographic characteristic	Category	*n*	%
Gender	Male	332	29.1
	Female	810	70.9
Type of institution	Double First-Class universities	105	9.2
	Public undergraduate institutions	990	86.7
	Private undergraduate institutions	37	3.2
	Higher vocational colleges	10	0.9
Geographic region of institution	East China	1,053	92.2
	South China	14	1.2
	Central China	19	1.7
	North China	15	1.3
	Northwest China	12	1.1
	Southwest China	10	0.9
	Northeast China	19	1.7
Academic year	Year 1 (Freshman)	353	30.9
	Year 2 (Sophomore)	202	17.7
	Year 3 (Junior)	228	20.0
	Year 4 (Senior)	79	6.9
	Year 5 and above	6	0.5
	Postgraduate	274	24.0
Disciplinary background	Philosophy	4	0.4
	Economics	21	1.8
	Law	30	2.6
	Education	56	4.9
	Literature	34	3.0
	History	2	0.2
	Natural sciences	157	13.7
	Engineering	227	19.9
	Agriculture	12	1.1
	Medicine	436	38.2
	Management	148	13.0
	Arts	15	1.3
Medical sub-discipline	Clinical medicine	14	1.2
	Stomatology	2	0.2
	Basic medical sciences	24	2.1
	Medical technology	9	0.8
	Nursing	263	23.0
	Traditional Chinese medicine / integrated medicine	4	0.4
	Public health and preventive medicine	92	8.1
	Pharmacy	24	2.1
	Other medicine-related	4	0.4
Political affiliation	Member of the Communist Party of China	147	12.9
	Member of the Communist Youth League	691	60.5
	Masses (non-affiliated)	302	26.4
	Other	2	0.2
Receipt of national or institutional financial aid	Student loan only	71	6.2
	Grant only	152	13.3
	Both loan and grant	102	8.9
	Neither	817	71.5
Prior visit to medicine-, anti-epidemic-, or public-health-themed exhibitions	Yes	648	56.7
	No	494	43.3

*N* = 1,142; *n* = frequency, % = percentage of the full sample. Medical sub-discipline categories are reported only for respondents whose disciplinary background was medicine; percentages are computed against the full sample (*N* = 1,142). Percentages may not sum to 100 due to rounding.

### 3.2 Measurement quality and descriptive statistics

Cronbach’s *α* values ranged from 0.928 to 0.965. The Kaiser–Meyer–Olkin value was 0.974 and Bartlett’s test of sphericity was statistically significant (*p* < 0.001), indicating that the item correlation matrix was suitable for exploratory factor analysis; factor loadings ranged from 0.612 to 0.857.

Harman’s single-factor test showed that the first unrotated factor accounted for 64.61% of the total variance, exceeding the prespecified 50% diagnostic threshold. Under this diagnostic rule, the result raises substantial concern about possible common method variance in the observed associations. The strong general factor may reflect shared method variance, a general evaluative response tendency, overlap among the study-defined constructs, socially desirable responding, or a combination of these influences.

To examine the discriminant validity among these four constructs and to further address potential concerns regarding common method bias, this study conducted confirmatory factor analysis (CFA) using SPSSAU software. Maximum likelihood estimation was employed during the analysis, and the discriminant validity of the variables was assessed by comparing the fit indices of different factor structure models.

First, a four-factor baseline model was specified in which Educational Cognition, Educational Emotion, DEC, and DER were represented as four distinct latent variables. The model showed good fit to the observed data: *χ*^2^/df = 1.802, RMSEA = 0.045, CFI = 0.966, TLI = 0.960, and SRMR = 0.048, indicating that the model fits the observed data well.

Second, to examine the relative adequacy of the four-factor structure, a three-factor competing model was specified by combining Educational Cognition and Educational Emotion into a single factor while retaining DEC and DER as separate factors. A one-factor model was also specified by loading all measurement items onto a single factor. Model fit deteriorated appreciably for the three-factor model (RMSEA = 0.059, CFI = 0.937) and was poorest for the one-factor model (RMSEA = 0.115, CFI = 0.677), which did not meet conventional standards for acceptable fit.

Taken together, the model-fit comparisons showed that the four-factor baseline model fit the data better than the selected three-factor and one-factor alternatives. These findings provide relative support for distinguishing Educational Cognition, Educational Emotion, DEC, and DER and indicate that the observed item covariance cannot be adequately represented by a single common factor. However, these model comparisons do not eliminate the concern about common method variance raised by the Harman test. The four constructs were therefore retained for the subsequent cross-sectional indirect-association analyses, whose results were interpreted cautiously.

Descriptive statistics for Educational Cognition, Educational Emotion, DER, and DEC are presented in Table 2.

**Table 2:** Descriptive statistics of the study variables.

Variable	*M*	SD
Educational Cognition	4.352	0.661
Educational Emotion	4.528	0.589
DER	4.306	0.677
DEC	4.370	0.682

*N* = 1,142.

Among the surveyed cohorts, Educational Emotion had the highest mean (*M* = 4.528). Both dimensions of PHLA also had relatively high means (DER = 4.306; DEC = 4.370), indicating comparatively strong reported aspirations for additional public-health-related learning within this sample.

### 3.3 Correlation analysis

Spearman’s rank-order correlation coefficients were calculated to examine the bivariate associations among Educational Cognition, Educational Emotion, DER, and DEC. The results are presented in Table 3.

**Table 3:** Spearman’s ρ correlation matrix of the study variables.

Variable	1	2	3	4
Educational Cognition	–			
Educational Emotion	0.649**	–		
DER	0.759**	0.664**	–	
DEC	0.733**	0.713**	0.826**	–

*N* = 1,142. Spearman’s ρ coefficients are reported. **p < 0.01.

As shown in Table 3, Educational Cognition was positively correlated with Educational Emotion (*ρ* = 0.649, *p* < 0.01), DER (*ρ* = 0.759, *p* < 0.01), and DEC (*ρ* = 0.733, *p* < 0.01). Educational Emotion was also positively correlated with DER (*ρ* = 0.664, *p* < 0.01) and DEC (*ρ* = 0.713, *p* < 0.01). The strongest correlation was observed between DER and DEC (*ρ* = 0.826, *p* < 0.01).

All study variables were significantly and positively related to one another, providing initial support for Hypothesis 1 (H1). The remaining bivariate associations provide a statistical basis for the subsequent cross-sectional indirect-association analyses.

### 3.4 Cross-sectional indirect-association analysis

**Figure 1:**
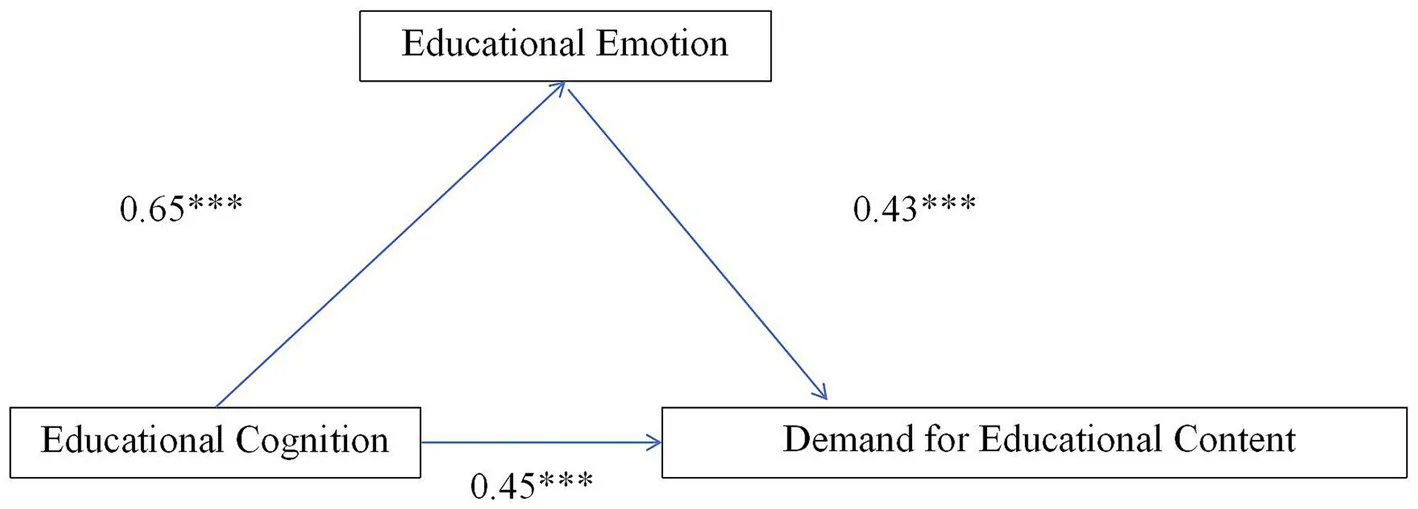
Cross-sectional indirect-association model involving Educational Cognition, Educational Emotion, and DER. Values are standardized regression coefficients (*β*). Arrows denote regression-model specification only and do not indicate temporal or causal direction. ***p < 0.001.

**Figure 2:**
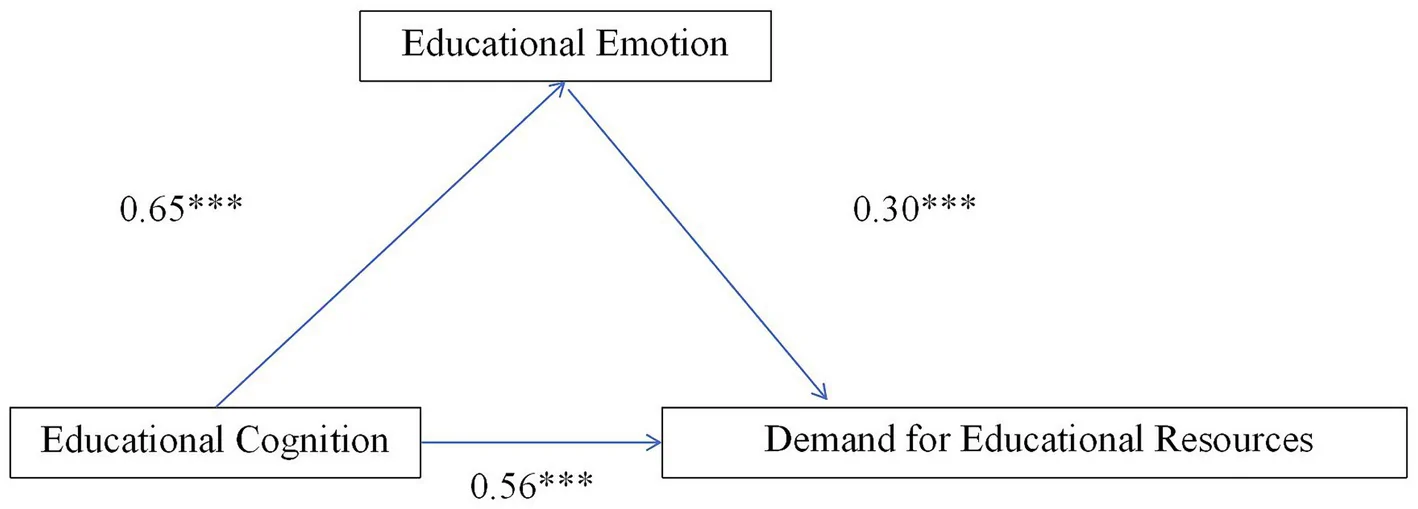
Cross-sectional indirect-association model involving Educational Cognition, Educational Emotion, and DEC. Values are standardized regression coefficients (*β*). Arrows denote regression-model specification only and do not indicate temporal or causal direction. ***p < 0.001.

Two cross-sectional indirect-association models were estimated using PROCESS Macro Version 3.3 (Model 4) for SPSS, with Educational Cognition specified as the focal predictor variable, Educational Emotion as the intervening statistical variable, and DER (H3a) or DEC (H3b) as separate outcome variables. All variables were standardized. These labels and the analytic ordering describe model specification only and do not imply causal or temporal ordering. Results are presented in Tables 4–7 and Figures 1, 2.

**Table 4:** Regression results for the cross-sectional Cognition–Emotion–DER indirect-association model.

Outcome variable	Predictor variable	*R*	*R* ^2^	*F*	*β*	*t*
Educational Emotion (Q)	Educational Cognition (R)	0.65	0.42	815.31***	0.65***	28.56
DER (Z)	Educational Cognition (R)	0.79	0.62	923.73***	0.56***	23.31
DER (Z)	Educational Emotion (Q)	–	–	–	0.30***	12.53

Standardized regression coefficients (β) are reported. Predictor and outcome labels denote statistical model specification only and do not imply causal or temporal ordering. ***p < 0.001.

**Table 5:** Standardized bootstrap estimates of the total, direct, and indirect effects in the cross-sectional Cognition–Emotion–DER indirect-association model.

Effect type	Standardized estimate	Boot SE	95% Bootstrap CI	Proportion of total effect
Total effect	0.75	0.02	[0.71, 0.79]	–
Direct effect	0.56	0.02	[0.51, 0.61]	74%
Indirect effect	0.20	0.02	[0.15, 0.23]	26%

Total, direct, and indirect effects are standard PROCESS output terms. Bootstrap confidence intervals were based on 5,000 resamples. An indirect effect was considered statistically different from zero when its 95% bootstrap CI excluded zero. These terms do not imply causal or temporal ordering.

**Table 6:** Regression results for the cross-sectional Cognition–Emotion–DEC indirect-association model.

Outcome variable	Predictor variable	*R*	*R* ^2^	*F*	*β*	*t*
Educational Emotion (Q)	Educational Cognition (R)	0.65	0.42	815.31***	0.65***	28.56
DEC (N)	Educational Cognition (R)	0.80	0.64	1008.48***	0.45***	19.30
DEC (N)	Educational Emotion (Q)	–	–	–	0.43***	18.50

Standardized regression coefficients (β) are reported. Predictor and outcome labels denote statistical model specification only and do not imply causal or temporal ordering. ***p < 0.001.

**Table 7:** Standardized bootstrap estimates of the total, direct, and indirect effects in the cross-sectional Cognition–Emotion–DEC indirect-association model.

Effect type	Standardized estimate	Boot SE	95% Bootstrap CI	Proportion of total effect
Total effect	0.73	0.02	[0.69, 0.77]	–
Direct effect	0.45	0.02	[0.41, 0.49]	61%
Indirect effect	0.28	0.02	[0.23, 0.33]	39%

Total, direct, and indirect effects are standard PROCESS output terms. Bootstrap confidence intervals were based on 5,000 resamples. An indirect effect was considered statistically different from zero when its 95% bootstrap CI excluded zero. These terms do not imply causal or temporal ordering.

#### 3.4.1 Cross-sectional indirect association involving educational emotion and DER

Educational Cognition was positively associated with Educational Emotion (*β* = 0.65, *p* < 0.001) and DER (*β* = 0.56, *p* < 0.001). Educational Emotion was also positively associated with DER (*β* = 0.30, *p* < 0.001).

The indirect association involving Educational Emotion was statistically different from zero for DER (estimate = 0.20, Boot SE = 0.02, 95% bootstrap CI = [0.15, 0.23]) and represented 26% of the total cross-sectional association (Table 5).

#### 3.4.2 Cross-sectional indirect association involving educational emotion and DEC

Educational Cognition was positively associated with Educational Emotion (*β* = 0.65, *p* < 0.001) and DEC (*β* = 0.45, *p* < 0.001). Educational Emotion was also positively associated with DEC (*β* = 0.43, *p* < 0.001).

The indirect association involving Educational Emotion was statistically different from zero for DEC (estimate = 0.28, Boot SE = 0.02, 95% bootstrap CI = [0.23, 0.33]) and represented 39% of the total cross-sectional association (Table 7).

#### 3.4.3 Comparison of the two cross-sectional indirect associations

Across the specified cross-sectional regression models, Educational Cognition was positively associated with Educational Emotion, DER, and DEC, supporting H2. The indirect-association estimate represented 26% of the total association for DER and 39% for DEC. These proportions came from separate outcome models and were not directly compared, so that the difference is descriptive and hypothesis-generating rather than confirmatory.

### 3.5 Sensitivity analyses by gender and disciplinary background

#### 3.5.1 Gender-stratified sensitivity analyses

The two cross-sectional indirect-association models were re-estimated separately for male and female students after adjustment for disciplinary background, institution type, and geographic region. For DER, the indirect-effect estimate involving Educational Emotion was statistically different from zero in both the male subgroup (estimate = 0.203, Boot SE = 0.046, 95% bootstrap CI [0.128, 0.283]) and the female subgroup (estimate = 0.198, Boot SE = 0.027, 95% bootstrap CI [0.146, 0.249]). For DEC, the corresponding estimate was also statistically different from zero among male students (estimate = 0.302, Boot SE = 0.046, 95% bootstrap CI [0.216, 0.394]) and female students (estimate = 0.282, Boot SE = 0.030, 95% bootstrap CI [0.226, 0.341]). The direction and magnitude of the estimates were broadly consistent across the two gender subgroups, and the confidence intervals overlapped. Subgroup-specific indirect-effect estimates are presented in Supplementary Table S2.

#### 3.5.2 Sensitivity analyses by disciplinary background

The models were also re-estimated separately for medical and non-medical students, with gender and the other prespecified demographic covariates included in the analyses. For DEC, the indirect-effect estimate involving Educational Emotion was statistically different from zero in both the non-medical subgroup (estimate = 0.334, Boot SE = 0.033, 95% bootstrap CI [0.270, 0.401]) and the medical subgroup (estimate = 0.242, Boot SE = 0.035, 95% bootstrap CI [0.177, 0.316]). For DER, the corresponding estimates were also statistically different from zero among non-medical students (estimate = 0.214, Boot SE = 0.030, 95% bootstrap CI [0.156, 0.273]) and medical students (estimate = 0.195, Boot SE = 0.031, 95% bootstrap CI [0.136, 0.261]). These findings indicate that the overall indirect-association pattern was not confined to either the medical or non-medical subgroup. Subgroup-specific indirect-effect estimates are presented in Supplementary Table S3.

## 4 Discussion

### 4.1 Interpretation of the cross-sectional associations

Educational Cognition, Educational Emotion, DER, and DEC were positively interrelated in the surveyed cohorts, and the indirect association involving Educational Emotion was statistically different from zero for both forms of stated learning demand. Within the CAC framework, this pattern is consistent with the co-occurrence of appraisal, affective-motivational response, and conative orientation. Because all variables were measured once, however, the framework should be understood as an organizing model rather than a demonstrated psychological sequence. The high correlation between DER and DEC (*ρ* = 0.826) supports their conceptual relatedness but also indicates substantial operational overlap; it does not, by itself, establish that the two domains form a single higher-order latent structure. Accordingly, the four study-defined domains should not be treated as sharply distinct operationalizations; associations involving them require extreme caution and should be regarded as preliminary in light of both construct overlap and possible common method variance.

The results are also compatible with integrated cognitive-affective accounts in which appraisals and emotional responses jointly relate to motivational orientations (23–25). The telemedicine study cited here provides only a conceptual parallel: it demonstrates the analytical value of considering cognitive and affective dimensions together, not equivalence between technology trust and historical education.

The descriptive DEC-DER contrast permits a tentative motivational interpretation. Self-Determination Theory suggests that demand for substantive content may be more closely connected to personal meaning and value internalization, whereas demand for media, sites, speakers, and delivery platforms may reflect the perceived utility of learning supports (26, 27). Control-Value Theory similarly treats value appraisals as central to educational emotion (28, 29), and a recent review describes emotion, cognition, motivation, and engagement as interconnected domains (30). These explanations remain *post hoc*: the study measured neither SDT constructs nor control-value appraisals, and the two indirect estimates were not directly compared. They should therefore be treated strictly as speculative and hypothesis-generating interpretations for future research, rather than as mechanisms supported by the present data.

Historical and professional narratives have been linked with identity, meaning-making, emotional alignment, professionalism, and collective engagement (31–37), while narrative-medicine scholarship provides an earlier conceptual basis for these connections (38). The present co-occurrence of favorable educational appraisals, affective-motivational responses, and stated learning demands is compatible with that literature, but it remains specific to the sampled IPE context.

The practical implication is therefore limited but relevant: public health history may be studied as both substantive educational content and a context in which appraisal, emotion, and stated demand are interrelated. The current estimates do not justify claims that a particular historical narrative or delivery format will cause greater engagement.

Future studies should preregister direct comparisons between DEC and DER, use validated and more clearly differentiated measures, assess autonomous motivation and control-value appraisals directly, and employ longitudinal or experimental designs that can establish temporal order. Behavioral indicators of course selection, resource use, or voluntary participation would also help distinguish stated demand from subsequent learning behavior.

The sensitivity analyses yielded broadly consistent indirect-association patterns across gender and disciplinary subgroups. The subgroup-specific estimates were statistically different from zero for both DER and DEC among male and female students and among medical and non-medical students. These findings suggest that the observed pattern was not confined to a single gender or disciplinary subgroup. However, the analyses were exploratory and did not constitute formal tests of between-group differences, measurement invariance, effect modification, or moderated mediation.

### 4.2 Limitations

First, the cross-sectional design precludes causal inference. Cross-sectional indirect-association analyses can yield biased estimates of longitudinal mediation parameters even under otherwise ideal conditions (39). The reported estimates therefore describe associations at one measurement occasion and do not demonstrate mediation over time.

Second, every focal variable was measured by self-report from the same respondents. The first factor in Harman’s single-factor test accounted for 64.61% of the total variance, exceeding the 50% diagnostic threshold; this finding raises substantial concern about possible common method variance in the observed associations (40). This concern is especially relevant in IPE because items involving national pride, patriotic values, collective responsibility, and endorsement of IPE may have recognizable preferred responses. The uniformly high scores may reflect not only favorable educational appraisals but also shared response tendencies, acquiescence, and socially desirable responding. Although anonymous administration, section separation, construct-specific wording, and partially differentiated response anchors may have reduced some response-related bias, no temporal or source separation, formal counterbalancing, marker variable, social-desirability measure, multi-informant assessment, or behavioral outcome was included. The CFA model comparison does not eliminate this concern. The reported correlations, regression coefficients, and indirect-association estimates may consequently be inflated and should be interpreted as preliminary and potentially upward-biased.

Third, the 24 substantive items were drawn from a self-developed 33-item questionnaire. EFA, internal-consistency estimates, and CFA model comparisons were reported. The four-factor model showed substantially better fit than the selected three-factor and one-factor alternatives, providing relative support for distinguishing Educational Cognition, Educational Emotion, DEC, and DER. Nevertheless, no measurement-invariance analysis was conducted, and no second-order model was tested; thus, the present results do not establish PHLA as a higher-order latent construct. The operational domains also partly overlap: Educational Cognition includes perceived emotional appeal; Educational Emotion includes affective responses, value endorsement, and volunteer willingness; and the DEC and DER items assess stated demands for educational content, resources, and delivery conditions rather than observed aspirations or behavior. This conceptual overlap may have contributed to the high intercorrelations and may limit the extent to which the four variables can be interpreted as sharply distinct constructs. Accordingly, the favorable CFA results should not be interpreted as eliminating the concern about common method variance indicated by the Harman test. Taken together, the high intercorrelations and the Harman result require extreme caution when interpreting the four scale scores as distinct construct operationalizations.

Finally, 92.2% of respondents attended institutions in Eastern China, 70.9% were female, and medicine was the largest disciplinary subgroup (38.2%), with nursing students particularly prominent. University names and cluster identifiers were not retained, and the analyses did not model within-class or within-institution dependence or apply sampling weights. Standard errors and confidence intervals may therefore be optimistic if responses were correlated within recruitment clusters. The findings apply to the surveyed cohorts and should not be extrapolated to all Chinese university students, non-Chinese populations, or educational settings outside IPE.

## 5 Conclusion

Educational Cognition, Educational Emotion, DER, and DEC were positively associated in the surveyed cohorts, and the cross-sectional indirect-association estimates involving Educational Emotion were statistically different from zero for both DER and DEC. These findings are associational and specific to the sampled IPE context; longitudinal studies are needed before any temporal, causal, or broader population-level interpretation can be made.

## Data Availability

The raw data supporting the conclusions of this article will be made available by the authors, without undue reservation.
