# Supplementary material for: Cognition, affect, and learning aspirations in public health development history education: cross-sectional associations in a large sample of university students in Eastern China
Source: Front Public Health. 2026 Aug 27;14:1921511. doi: 10.3389/fpubh.2026.1921511 (PMC13558392; doi:10.3389/fpubh.2026.1921511)
Supplement: Supplementary file 1 [file Table_1.DOC]

1. **Supplementary Table S1**

**Variables Included in the Analysis and Their Coding**

| **Variable / Item** | **Response Format and Coding** |
| --- | --- |
| Gender | Male = 1; Female = 2 |
| Type of institution | Double First-Class university = 1; Public undergraduate institution = 2; Private undergraduate institution = 3; Higher vocational college = 4 |
| Geographic region of the institution | East China (Shanghai, Jiangsu, Zhejiang, Anhui, Fujian, Jiangxi, and Shandong) = 1; South China (Guangdong, Guangxi, and Hainan) = 2; Central China (Hubei, Hunan, and Henan) = 3; North China (Beijing, Tianjin, Hebei, Shanxi, and Inner Mongolia) = 4; Northwest China (Shaanxi, Gansu, Qinghai, Ningxia, and Xinjiang) = 5; Southwest China (Sichuan, Chongqing, Guizhou, Yunnan, and Tibet) = 6; Northeast China (Liaoning, Jilin, and Heilongjiang) = 7 |
| Current year of study | First-year undergraduate = 1; Second-year undergraduate = 2; Third-year undergraduate = 3; Fourth-year undergraduate = 4; Fifth year or above = 5; Postgraduate = 6 |
| Academic discipline | Philosophy = 1; Economics = 2; Law = 3; Education = 4; Literature = 5; History = 6; Natural sciences = 7; Engineering = 8; Agriculture = 9; Medicine = 10; Management = 11; Arts = 12 |
| If you are enrolled in a medical discipline, what is your medical sub-discipline? | Clinical medicine = 1; Stomatology = 2; Basic medical sciences = 3; Medical technology = 4; Nursing = 5; Traditional Chinese medicine / integrated traditional Chinese and Western medicine = 6; Public health and preventive medicine = 7; Pharmacy = 8; Other medicine-related disciplines = 9 |
| Political affiliation | Member of the Communist Party of China = 1; Member of the Communist Youth League = 2; Non-affiliated (masses) = 3; Other = 4 |
| Have you received student financial aid provided by the government or your institution? | Student loan only = 1; Grant only = 2; Both loan and grant = 3; Neither = 4 |
| Prior visit to medicine-, anti-epidemic-, or public-health-themed exhibitions | Yes=1, No=2 |
| **Educational Emotion** |  |
| Studying the history of China's public health development helps strengthen national pride and national identification. | Strongly disagree = 1; Disagree = 2; Neutral = 3; Agree = 4; Strongly agree = 5 |
| The patriotic spirit embedded in the history of public health development is worthy of being inherited and promoted. | Strongly disagree = 1; Disagree = 2; Neutral = 3; Agree = 4; Strongly agree = 5 |
| Universities should incorporate this type of historical education as an important component of Ideological and Political Education. | Strongly disagree = 1; Disagree = 2; Neutral = 3; Agree = 4; Strongly agree = 5 |
| I am willing to participate in public-health-related volunteer services, such as health education and free-clinic activities. | Strongly disagree = 1; Disagree = 2; Neutral = 3; Agree = 4; Strongly agree = 5 |
| I am proud of the achievements in the development of China's public health enterprise. | Strongly disagree = 1; Disagree = 2; Neutral = 3; Agree = 4; Strongly agree = 5 |
| **Demand for Educational Content** |  |
| School courses or activities place emphasis on identifying the Ideological and Political Education elements embedded in the history of public health development. | Strongly disagree = 1; Disagree = 2; Neutral = 3; Agree = 4; Strongly agree = 5 |
| School courses or activities systematically introduce the development of public health in China across different historical periods. | Strongly disagree = 1; Disagree = 2; Neutral = 3; Agree = 4; Strongly agree = 5 |
| School courses or activities present the institutional, scientific and technological, and organizational innovations achieved by China during public health crises. | Strongly disagree = 1; Disagree = 2; Neutral = 3; Agree = 4; Strongly agree = 5 |
| The educational content covers the contributions of diverse actors, including scientists, frontline healthcare workers, public health administrators, and community workers. | Strongly disagree = 1; Disagree = 2; Neutral = 3; Agree = 4; Strongly agree = 5 |
| The educational content maintains coherence and consistency across different courses or activities. | Strongly disagree = 1; Disagree = 2; Neutral = 3; Agree = 4; Strongly agree = 5 |
| **Demand for Educational Resources** |  |
| The institution provides sufficient media resources, such as documentaries, courseware, and historical footage. | Strongly disagree = 1; Disagree = 2; Neutral = 3; Agree = 4; Strongly agree = 5 |
| The institution collaborates with practice bases or memorial venues, such as medical museums and 'red' education bases. | Strongly disagree = 1; Disagree = 2; Neutral = 3; Agree = 4; Strongly agree = 5 |
| Frontline public health practitioners, such as CDC personnel and healthcare workers involved in epidemic response, are invited to share their experiences. | Strongly disagree = 1; Disagree = 2; Neutral = 3; Agree = 4; Strongly agree = 5 |
| New media, such as WeChat official accounts and short-video platforms, are used to publicize exemplary individuals and their deeds. | Strongly disagree = 1; Disagree = 2; Neutral = 3; Agree = 4; Strongly agree = 5 |
| Instructors possess relevant disciplinary knowledge and humanistic literacy and can explain historical events and their underlying values in depth. | Strongly disagree = 1; Disagree = 2; Neutral = 3; Agree = 4; Strongly agree = 5 |
| Educational formats are diverse, such as thematic lectures, film-viewing seminars, situational dramas, and social practice. | Strongly disagree = 1; Disagree = 2; Neutral = 3; Agree = 4; Strongly agree = 5 |
| The teaching process is lively and engaging and can stimulate learning interest and reflection. | Strongly disagree = 1; Disagree = 2; Neutral = 3; Agree = 4; Strongly agree = 5 |
| Students have opportunities to choose learning approaches that suit them, such as online learning, field visits, and group projects. | Strongly disagree = 1; Disagree = 2; Neutral = 3; Agree = 4; Strongly agree = 5 |
| Interaction and discussion are encouraged during teaching to enhance students' sense of participation. | Strongly disagree = 1; Disagree = 2; Neutral = 3; Agree = 4; Strongly agree = 5 |
| Feedback channels for institutional educational activities are accessible, allowing students' views to be heard. | Strongly disagree = 1; Disagree = 2; Neutral = 3; Agree = 4; Strongly agree = 5 |
| **Educational Cognition: Performance of Ideological and Political Education in the History of Public Health Development** |  |
| Content authenticity | Very poor = 1; Poor = 2; Average = 3; Good = 4; Very good = 5 |
| Emotional appeal | Very poor = 1; Poor = 2; Average = 3; Good = 4; Very good = 5 |
| Capacity for value guidance | Very poor = 1; Poor = 2; Average = 3; Good = 4; Very good = 5 |
| Practical inspiration | Very poor = 1; Poor = 2; Average = 3; Good = 4; Very good = 5 |

2. **Supplementary Table S2**

**Gender-stratified sensitivity analyses of the cross-sectional indirect-association models**

| **Outcome and effect type** | **Male students Estimate** | **Boot SE** | **95% bootstrap CI** | **Female students Estimate** | **Boot SE** | **95% bootstrap CI** |
| --- | --- | --- | --- | --- | --- | --- |
| **DER** |  |  |  |  |  |  |
| Indirect effect | 0.203 | 0.046 | [0.128, 0.283] | 0.198 | 0.027 | [0.146, 0.249] |
| Direct effect | 0.580 | 0.051 | [0.487, 0.672] | 0.567 | 0.029 | [0.510, 0.624] |
| **DEC** |  |  |  |  |  |  |
| Indirect effect | 0.302 | 0.046 | [0.216, 0.394] | 0.282 | 0.030 | [0.226, 0.341] |
| Direct effect | 0.464 | 0.049 | [0.368, 0.559] | 0.462 | 0.028 | [0.408, 0.516] |

*Note.* Gender-stratified models were adjusted for disciplinary background, institution type, and geographic region. Direct and indirect effects are standard PROCESS output terms and do not imply causal or temporal ordering. Subgroup sizes were male n = 332 and female n = 810.

3. **Supplementary Table S3**

**Sensitivity analyses stratified by medical versus non-medical disciplinary background**

| **Outcome** | **Non-medical students Estimate** | **Boot SE** | **95% bootstrap CI** | **Medical students Estimate** | **Boot SE** | **95% bootstrap CI** |
| --- | --- | --- | --- | --- | --- | --- |
| DEC: Indirect effect | 0.334 | 0.033 | [0.270, 0.401] | 0.242 | 0.035 | [0.177, 0.316] |
| DER: Indirect effect | 0.214 | 0.030 | [0.156, 0.273] | 0.195 | 0.031 | [0.136, 0.261] |

*Note.* Discipline-stratified models were adjusted for gender, institution type, and geographic region. Indirect effect is a standard PROCESS output term and does not imply causal or temporal ordering. Subgroup sizes were non-medical n = 706 and medical n = 436.
